# The epidemiology of metabolic dysfunction-associated steatotic liver disease among pediatric patients with type 2 diabetes: Systematic review and meta-analysis

**DOI:** 10.1007/s00431-025-06734-0

**Published:** 2026-02-05

**Authors:** Letícia Rocha Campos, Samira Mohamad Khalil, Matheus Souza

**Affiliations:** 1 Department of Medicine, University of Ribeirão Preto, Ribeirão Preto, São Paulo Brazil; 2 Department of Medicine, Universidad de Buenos Aires, Buenos Aires, Argentina; 3 Department of Internal Medicine, Federal University of Rio de Janeiro, 255 Professor Rodolpho Paulo Rocco Av, Rio de Janeiro, 21941‑913 RJ Brazil

**Keywords:** Non-alcoholic fatty liver disease, NAFLD, Burden, MASLD, MASH, Metabolism, Diabetes mellitus

## Abstract

Metabolic dysfunction-associated steatotic liver disease (MASLD) is strongly associated with type 2 diabetes (T2D) in adults; however, its epidemiology in the pediatric population remains unclear. We conducted a systematic review and meta-analysis to estimate the prevalence of MASLD among children and adolescents with T2D. We systematically searched PubMed and Embase databases from inception until March 18, 2025 to identify observational studies investigating the prevalence of MASLD (diagnosed by liver biopsy, imaging methods, or blood-based biomarkers) in children and adolescents (aged ≤ 21 years) with T2D. Data from eligible studies were extracted, and meta-analysis was performed using a generalized linear mixed model. This study was registered in PROSPERO (ID CRD420251013625). Eighteen unique studies with 3926 pediatric patients with T2D were included. The pooled prevalence of MASLD in pediatric T2D was 36.61% (95% confidence interval [CI] 26.45 to 48.12), with substantial heterogeneity (I^2^ = 97.1%). Prevalence estimates differed significantly by the diagnostic method used for MASLD (p = 0.014) but remained consistent across subgroups based on world region, median year of enrollment, and sample size. Sensitivity analysis restricted to magnetic resonance-based studies showed a high prevalence (55.0%, 95% CI 38.20 to 70.78, I^2^ = 73.7%). The funnel plot did not reveal any significant publication bias.

*Conclusions*: MASLD affects over one-third of pediatric patients with T2D. These findings support early liver health screening in this high-risk group. Future research is needed to validate non-invasive tests for liver disease assessment in pediatric diabetes care.

**Table:** 

**What is known:** • *Type 2 diabetes (T2D) is a strong risk factor for metabolic dysfunction-associated steatotic liver disease (MASLD).* • *The epidemiology of MASLD in children and adolescents with T2D remains poorly characterized.*
**What is new:** • *MASLD affects an estimated 36.61% of pediatric patients with T2D, with substantial variation according to the diagnostic modality used.* • *This high prevalence underscores the need for early liver health screening in pediatric T2D.*

**Supplementary Information:**

The online version contains supplementary material available at 10.1007/s00431-025-06734-0.

## Introduction

Metabolic dysfunction-associated steatotic liver disease (MASLD), formerly known as non-alcoholic fatty liver disease (NAFLD), has become the most common form of chronic liver disease in children and adolescents, coinciding with the rise in childhood obesity [1]. Although this condition is multifactorial, one significant component includes lifestyle and dietary changes, involving sedentary behavior and increased consumption of unhealthy foods, such as hypercaloric diets high in saturated fats, sugars, fructose-containing beverages, and ultra-processed products [1–4]. MASLD affects an estimated 7%−13% of the general pediatric population and 47%−52% of individuals with obesity [5, 6], with a considerable number of these cases already presenting advanced liver disease [7]. MASLD is particularly concerning because it can lead to liver-related complications, such as cirrhosis and hepatocellular carcinoma. It is also associated with extrahepatic complications in adulthood, including cardiovascular disease, chronic kidney disease, and extrahepatic cancers. [8–11].

Type 2 diabetes (T2D) is a strong predictor of MASLD, and the two conditions share a bidirectional and synergistic relationship driven by chronic inflammation and insulin resistance [12–15]. While this association has been extensively studied in adults, the underlying pathophysiology and epidemiology of MASLD in youth with T2D remain unclear [16, 17]. Notably, the literature has documented the growing prevalence and incidence of youth-onset T2D, which is characterized by an aggressive course of diabetes and its complications. However, there is a lack of comprehensive synthesis of the available evidence on the extent of the MASLD burden in this high-risk group, which is necessary to inform future screening and management strategies. To address this knowledge gap, we conducted a systematic review and meta-analysis to evaluate the prevalence of MASLD in children and adolescents with T2D and to explore potential sources of heterogeneity.

## Methods

### Protocol registration

We followed the Meta-analysis Of Observational Studies in Epidemiology (MOOSE) reporting guidelines [18]. The study protocol was a priori registered in the International Prospective Register of Systematic Reviews (PROSPERO) database (ID CRD420251013625).

### Search strategy and study selection

A comprehensive search of PubMed and Embase databases was performed from inception to March 18, 2025. The full search strategy is described in detail in the **Supplementary Methods**. Two authors (LRC and SMK) independently screened titles and abstracts identified in the primary search and further assessed full-text articles based on eligibility criteria. Disagreements were resolved in consultation with a third author (MS). We included studies if they reported data on the prevalence of MASLD (detected by liver biopsy, imaging, blood-based biomarkers, or liver enzymes) in pediatric patients (aged ≤ 21 years as defined by the FDA [19]) diagnosed with T2D. Studies using older NAFLD terminology and diagnostic frameworks were also included [20], acknowledging that historical liver enzyme-based definitions may not fully align with current MASLD criteria; this potential discrepancy was addressed through a planned sensitivity analysis. We excluded studies that focused on special populations and included adult patients. To reduce small-study effects and avoid unstable prevalence estimates, we excluded studies with small sample sizes (i.e., < 20 patients). In addition, we excluded reviews, meta-analyses, abstracts, editorials, case reports, protocols, randomized controlled trials, and commentaries. In the case of overlapping populations, we included only the study with the most comprehensive data.

### Data collection and quality assessment

Data were extracted using a standardized extraction form. The following data were extracted for each study: (1) study characteristics: first author, location, study design, study setting, enrollment period, diagnosis of MASLD; (2) population characteristics: sample size, age, sex, body mass index (BMI), HbA1c; and (3) prevalence of MASLD. The transformation described by Wan et al. [21] was used to convert data presented as median (range or interquartile range) to mean (standard deviation). The risk of bias of the included studies was performed independently by two authors (LRC and SMK) using the tool developed by Hot et al. [22] to assess the methodological quality of a prevalence study. Disagreements were resolved in consultation with a third author (MS). We assigned a score of 1 as low risk of bias and 0 as high risk of bias, with overall risk of bias further categorized as low (≥ 9), moderate (8–4), or high (≤ 3).

### Statistical analysis

The primary outcome of this study was the prevalence of MASLD in the pediatric T2D population. Statistical analysis was performed using R software (*“meta”* and *“metafor”* packages; version 4.2.3); a p-value < 0.05 defined statistical significance. Meta-analysis of proportions was performed using a generalized linear mixed model with Clopper-Pearson intervals. Heterogeneity was assessed using the I^2^ statistic, with values of ~ 25%, ~ 50%, and ~ 75% representing low, moderate, and high heterogeneity, respectively; significant heterogeneity was defined as I^2^ > 50% [23]. To explore the sources of heterogeneity in the prevalence of MASLD, we performed subgroup analyses according to study design, location, average age, and diagnostic method of MASLD. We then performed meta-regressions based on study-level variables, such as female proportion, age and BMI. Sensitivity analyses were conducted by restricting the analysis to (1) studies using imaging-based diagnosis and (2) those using magnetic resonance-based techniques for the diagnosis of MASLD. Publication bias was assessed by visual inspection of the funnel plot, as there is no established tool for assessment in single-arm meta-analyses.

## Results

### Summary of included studies

**Fig. 1:**
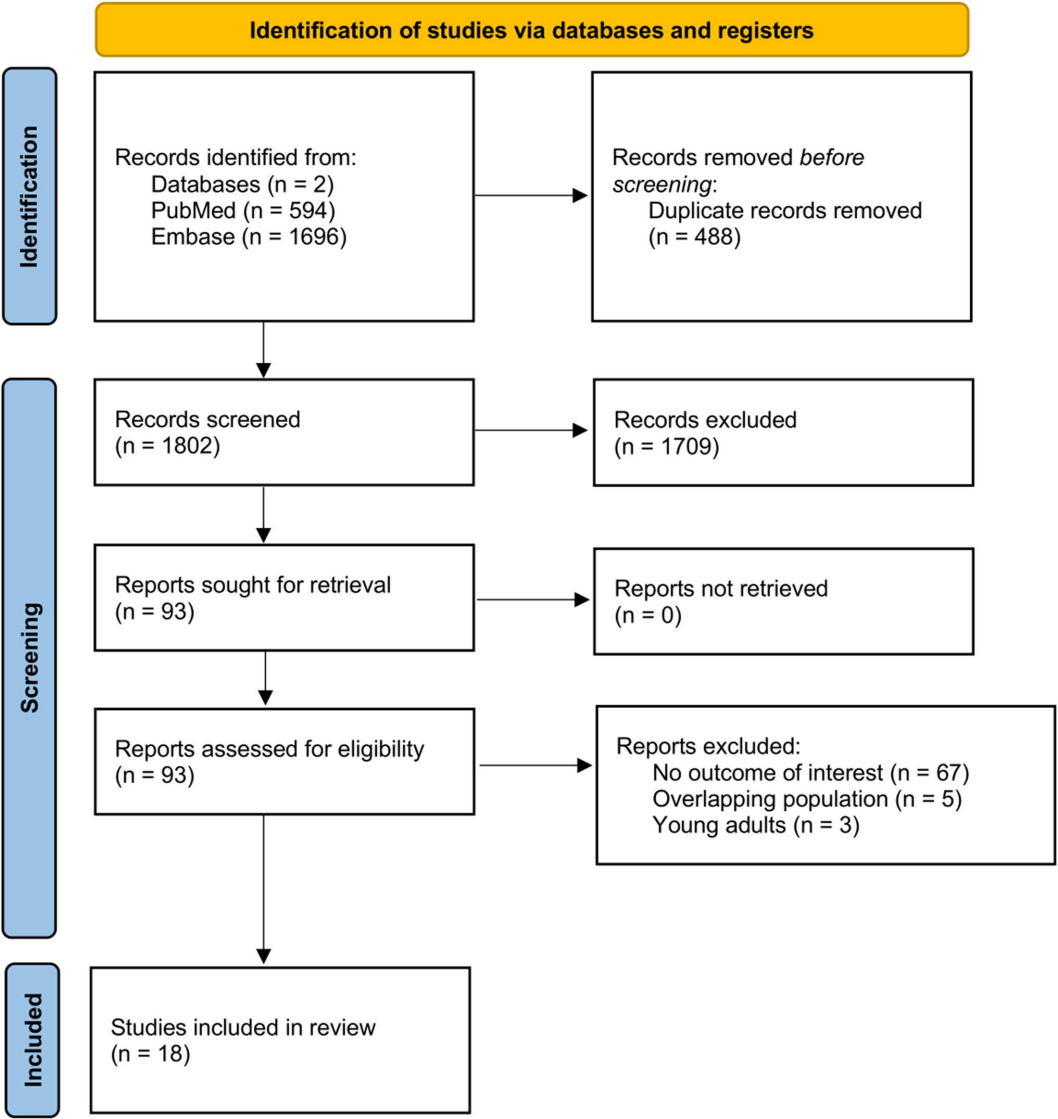
PRISMA flow diagram of study screening and selection. PRISMA, Preferred Reporting Items for Systematic Reviews and Meta-Analyses

We identified 2290 records through our initial search strategy. After excluding duplicates and ineligible studies, we assessed 93 potentially eligible studies for inclusion. Ultimately, a total of 18 studies involving 3926 pediatric patients with T2D were included in the meta-analysis (Fig. 1) [24–41].

Table 1 summarizes the main characteristics of the included studies. Of these, 9 studies were conducted in North America, 5 in Asia, 2 in Oceania, 1 in Europe, and 1 in Africa. There were 10 single-center studies and 8 multicenter studies. The diagnosis of MASLD in the pediatric population was made based on high liver enzymes only (n = 6), high liver enzymes or ultrasound (n = 4), magnetic resonance spectroscopy (MRS) (n = 2), magnetic resonance imaging (MRI) (n = 1) or ultrasound (n = 1). Of note, 4 studies did not report the diagnostic method used for MASLD. All studies were conducted in health-care centers in a time frame between 1995–2020. The risk of bias was low in the included studies (Fig. S1).

**Table 1:** Main characteristics of the included studies

First author (year)	Study characteristics					Population characteristics				
	Country	Study enrollment	Design	Center	Diagnostic method of MASLD	Sample size	Female, %	Age, years	BMI, kg/m^2^ (or BMI SDS)	HbA1c.%
Nadeau et al. (2005) [25]	United States	1996–2002	Retrospective cohort	Single center	High liver enzymes	48	56.3	15 (0.6)	33.1 (1)	8.5 (0.3)
Eppens et al. (2006) [26]	Australia	1996–2005	Cross-sectional	Single center	High liver enzymes	68	50.0	13.2 [11.6–15.0]	BMI SDS 1.86 [1.28–2.40]	7.3 [6.0–8.3]
Jin et al. (2011) [31]	China	-	Retrospective cohort	Single center	High liver enzymes or ultrasound	31	29.0	12.5 (1.6)	-	-
Wittmeier et al. (2012) [27]	Canada	-	Cross-sectional	Single center	Magnetic resonance spectroscopy	27	59.3	15 (1.5)	BMI SDS 2.0 (0.5)	7.2 (1.6)
Jefferies et al. (2012) [24]	New Zealand	1995–2007	Prospective cohort	Multicenter	High liver enzymes	52	-	12.9 (1.8)	BMI SDS 2.3 (0.4)	9.5 (2.5)
Hudson et al. (2012) [28]	United States	2005–2006	Retrospective cohort	Single center	High liver enzymes	57	47.4	Mean 13.3	Mean 35.8	Mean 8.9
Fu et al. (2013) [29]	China	1995–2010	Retrospective cohort	Multicenter	Unclear	349	42.1	-	-	-
Osman et al. (2013) [30]	Sudan	2006–2009	Cross-sectional	Single center	High liver enzymes	38	-	-	-	-
Candler et al. (2018) [33]	United Kingdom and Republic of Ireland	2015–2016	Prospective cohort	Multicenter	Unclear	106	67.0	14.3 {7.9–16.9}	BMI SDS 2.89	-
Cree-Green et al. (2019) [32]	United States	-	Cross-sectional	Single center	Magnetic resonance imaging	27	77.8	15.6 (0.2)	31.2 [28.5–38.8]	7.3 [6.2–10.1]
Tung et al. (2021) [34]	Hong Kong	2008–2017	Retrospective cohort	Multicenter	High liver enzymes or ultrasound	391	51.9	14.7 (2.1)	BMI SDS 2.28 (1.03)	-
Astudillo et al. (2021) [35]	United States	2016–2019	Retrospective cohort	Single center	Unclear	376	66.0	13.6 (2.5)	BMI SDS 2.22	9.5
Ahmed et al. (2021) [36]	Qatar	2018–2020	Prospective cohort	Multicenter	Ultrasound	104	44.2	13.87 (2.6)	BMI SDS 2.43 (0.56)	10.07 (2.35)
Bacha et al. (2022) [37]	United States	2012–2018	Cross-sectional	Multicenter	Unclear	1217	62.9	Mean 13.4	BMI SDS 2.3	9.9
Beauchamp et al. (2021) [38]	United States	2004–2016	Retrospective cohort	Single center	High liver enzymes	151	72.2	14 (2)	BMI 36.8 (7.6); BMI SDS 2.4 (0.4)	10.3 (2.5)
Levin et al. (2022) [40]	Israel	2008–2019	Retrospective cohort	Multicenter	High liver enzymes or ultrasound	379	60.2	14.7 (1.9)	32.7 (7.8); BMI SDS 1.96 (0.77)	8.8 (2.6)
Patel et al. (2023)—Cohort 1 [39]	Canada	2006–2008	Prospective cohort	Multicenter	High liver enzymes or ultrasound	176	-	-	-	-
Patel et al. (2023)—Cohort 2 [39]		2017–2019				282	-	-	-	-
Morales et al. (2024) [41]	Mexico	-	Cross-sectional	Single center	Magnetic resonance spectroscopy	47	74.5	Mean 15.6	BMI 25.1; BMI SDS 1.2	7.9

Continuous data are reported as mean (standard deviation), median [interquartile range] or median {range}

Abbreviations: *BMI* body mass index; *MASLD* metabolic dysfunction-associated steatotic liver disease

### Prevalence of MASLD in pediatric patients with T2D

**Fig. 2:**
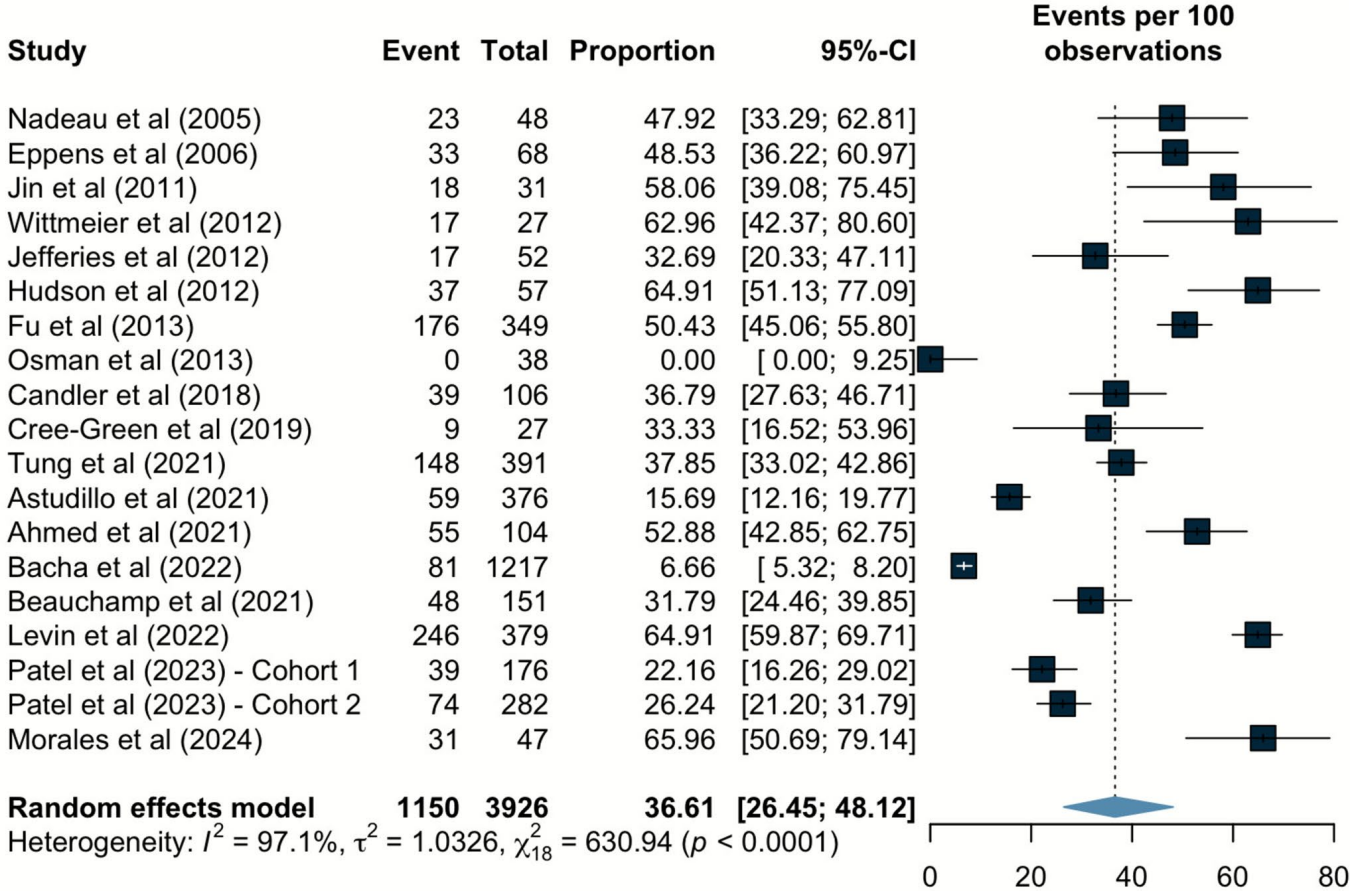
Forest plot reporting the prevalence of MASLD in pediatric population with type 2 diabetes. MASLD, metabolic dysfunction-associated steatotic liver disease; CI, confidence interval

The estimated pooled prevalence of MASLD in pediatric T2D was 36.61% (95% CI 26.45 to 48.12) from 18 studies, with high heterogeneity (I^2^ = 97.1%) (Fig. 2).

Subgroup analyses were performed to stratify MASLD prevalence and identify possible sources of high heterogeneity across the included studies (Table 2). No significant differences were found by world region (p = 0.12), average age (p = 0.11), center (p = 0.67), median study year of enrollment (p = 0.22), or sample size (p = 0.59). However, significant differences were found when stratified by diagnostic method (p = 0.014), with prevalence rates of MASLD in high liver enzymes only, high liver enzymes or ultrasound, MRS, MRI, and ultrasound estimated to be 31.89% (95% CI 13.10 to 59.25, I^2^ = 76.7%), 40.40% (95% CI 26.15 to 56.47, I^2^ = 97.1%), 64.86% (95% CI 53.39 to 74.85, I^2^ = 0%), 33.33% (95% CI 16.52 to 53.96, I^2^ = NA), and 52.88% (95% CI 42.85 to 62.75, I^2^ = NA), respectively. The pooled prevalence of MASLD in people with T2D, as determined by studies that used imaging for diagnosis, was 54.55% (95% CI 43.76 to 64.93, I^2^ = 62%; Fig. S2). Restricting the analysis to studies with an MR-based MASLD diagnosis yielded an estimated pooled prevalence of 55.03% (95% CI 38.20 to 70.78, I^2^ = 73.7%; Fig. S3).

**Table 2:** Overall and stratified prevalence of MASLD in pediatric population with type 2 diabetes

	Studies, *n*	Patients, *n*	Pooled prevalence, %	95% confidence interval	I^2^, %	*p-*value
Overall	18	3926	36.61	26.45 to 48.12	97.1	-
World region						
Asia	5	1254	52.35	43.41 to 61.15	92.8	0.12
Africa	1	38	No cases	-	-	
Europe	1	106	36.79	27.63 to 46.71	-	
North America	9	2408	33.86	21.10 to 49.50	96.8	
Oceania	2	120	41.22	30.71 to 52.60	66.8	
Average age						
< 15 years	11	2932	37.92	25.65 to 51.95	98.2	0.11
≥ 15 years	4	149	53.16	40.69 to 65.25	65.1	
Center						
Single-center	10	870	38.89	22.98 to 57.58	92.4	0.67
Multicenter	8	3056	33.98	22.14 to 48.23	98.4	
Median study year of enrollment						
< 2015	10	1709	37.53	24.06 to 53.25	92.8	0.22
≥ 2015	5	2085	23.67	11.88 to 41.65	98	
Sample size						
< 100	9	395	42.82	25.64 to 61.93	61.2	0.59
100–300	4	819	32.95	24.58 to 42.57	87.7	
> 300	5	2712	30.19	13.33 to 54.89	99.2	
Diagnostic method						
High liver enzymes	6	414	31.89	13.10 to 59.25	76.7	0.014
High liver enzymes or ultrasound	4	1259	40.40	26.15 to 56.47	97.1	
Magnetic resonance spectroscopy	2	74	64.86	53.39 to 74.85	0	
Magnetic resonance imaging	1	27	33.33	16.52 to 53.96	-	
Ultrasound	1	104	52.88	42.85 to 62.75	-	
Sensitivity analysis						
Only imaging studies	4	205	54.55	43.76 to 64.93	62	-
Only magnetic resonance-based diagnosis	3	101	55.03	38.20 to 70.78	73.7	-

*MASLD* metabolic dysfunction-associated steatotic liver disease

**Fig. 3:**
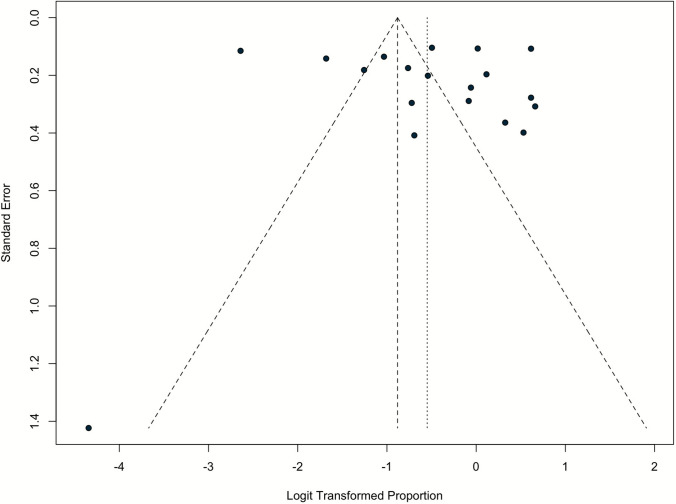
Funnel plot evaluating the possibility of publication bias across the eligible studies

Meta-regressions showed no impact of study-level moderators on MASLD prevalence, including female proportion (p = 0.16), mean age (p = 0.30) and mean BMI-SDS (p = 0.10) (Table S1). The visual inspection of the funnel plot shows a symmetrical distribution of studies around the pooled effect size, indicating no apparent small-study effects (Fig. 3). This visual assessment is supported by Egger’s regression test (p = 0.64), which quantitatively confirms the absence of significant publication bias in the meta-analysis.

## Discussion and conclusions

### Main findings

In this systematic review and meta-analysis involving 18 studies and 3926 children, we estimated the burden of MASLD in pediatric population with T2D. We found that the pooled prevalence of MASLD was approximately 37%, with high heterogeneity partly driven by the diagnostic method. Notably, prevalence estimates ranged from 31.9% when defined by elevated liver enzymes alone to 40.4% with enzymes or ultrasound, 52.9% with ultrasound, 33.3% with MRI, and 64.9% with MRS. Overall, these estimates remained consistent when stratified by other subgroups, and meta-regression analysis suggested no significant influence of sex, age, or BMI-SDS on MASLD prevalence.

### In the context of current evidence

Given its high prevalence and impact on the severity of liver disease, there is increasing recognition of the importance of monitoring liver health in adults with T2D [13, 42]. However, with the rising incidence of young-onset T2D alongside childhood obesity [16], understanding the epidemiology and risk factors of MASLD is also critical to developing early screening and management strategies for this group. Not surprisingly, we found a lower prevalence of MASLD compared to the ~ 70% prevalence found in T2D adults [42]. This difference is likely due to the shorter duration of diabetes and lower exposure to unhealthy lifestyles. In addition, genetic and epigenetic susceptibility (e.g., *PNPLA3* variants, methylation changes), differences in visceral adiposity distribution, and more severe insulin resistance phenotypes may accelerate intrahepatic fat accumulation and contribute to the heterogeneity of MASLD expression in youth with T2D early in life [43–46]. These factors may also shape disease penetrance and progression, reinforcing the importance of early identification and personalized risk stratification.

The childhood period represents a crucial window for early care, given the potential for prolonged diabetes duration and the associated progression to advanced liver disease and complications in adulthood. Current recommendations for MASLD screening in T2D are focused on the adult population [13, 47], and there is limited discussion on applying these strategies to youth [48]. Existing recommendations from AASLD and AAP are primarily based on age and overweight/obesity in addition to the presence of cardiometabolic risk factors (e.g., T2D) [49, 50]. The International Society for Pediatric and Adolescent Diabetes (ISPAD) guidelines recommend screening with alanine aminotransferase at the time of T2D diagnosis in children and adolescents [51], though broader implementation of systematic MASLD screening strategies in this population remains limited. Notably, the absence of consolidated non-invasive diagnostic tools for evaluating liver disease severity in children — which were typically used for NAFLD before the updated diagnostic criteria for the current term (MASLD) were established — highlights a significant gap in clinical knowledge/practice between adult and pediatric care.

Our results also suggest that the burden of MASLD is higher in the pediatric T2D population than in the pediatric type 1 diabetes (T1D) population [52]. A recent meta-analysis by Moura et al. [52] estimated a MASLD prevalence of ~ 17% based on nine studies involving 1007 children and adolescents with T1D. This discrepancy aligns with findings in the adult population and may be attributed to the differences in pathophysiology of T1D and T2D [42, 53]. Although T2D is less prevalent among youth, it is worth emphasizing that its systemic metabolic milieu, characterized by insulin resistance, obesity, and low-grade inflammation, contributes more directly to liver steatosis and subsequent progression of MASLD [14]. In contrast, T1D is primarily an autoimmune disease involving insulin deficiency; nonetheless, MASLD occurrence in this population is likely driven by different mechanisms, such as poor glycemic control and coexisting cardiometabolic risk factors [54], with a potential protective role of insulin therapy [53, 55].

### Limitations

This meta-analysis has important limitations, some of which are inherent in the nature of the included studies. First, we found high I^2^ in our estimates, indicating substantial between-study variability, which we were able to partially account for in subgroup analyses. In addition, our meta-regressions did not identify significant effects of plausible moderators (e.g., sex, age, and BMI-SDS) on MASLD prevalence. These findings are more likely driven by limitations in data granularity, inconsistent reporting, and methodological heterogeneity across studies than by true biological consistency and should therefore be interpreted with caution. Second, given the invasive nature of liver biopsy, most studies included MASLD diagnosed by non-invasive methods, mainly liver enzymes, as recommended for the diagnosis of suspected MASLD. In particular, liver enzymes and ultrasound, although widely available, have limited sensitivity for detecting mild steatosis and provide only indirect or qualitative information on hepatic steatosis. In contrast, MRI-based techniques provide accurate, quantitative evaluations of intrahepatic fat content, generally yielding higher prevalence estimates. Consequently, studies that employed these less precise methods may have underestimated MASLD prevalence, contributing to the observed heterogeneity across studies. Third, the predominance of tertiary or hospital-based cohorts may have introduced selection bias, limiting the generalizability of our findings to community/population-based. Specifically, children and adolescents who are treated at specialized diabetes or hepatology centers may have more severe disease and a higher metabolic risk than those treated in community/population-based settings. Fourth, several of the included studies used the previous NAFLD nomenclature, with some defining the disease based solely on elevated liver enzymes — criteria that no longer align with the updated pediatric MASLD definition, which requires objective evidence of hepatic steatosis in conjunction with at least one cardiometabolic risk factor (e.g., T2D). In accordance with systematic review principles, we did not reinterpret historical studies using contemporary criteria. Instead, we adopted a comprehensive approach and examined robustness through imaging-restricted sensitivity analysis, which showed a high MASLD prevalence. Finally, data were limited for estimation of the prevalence of MASH and advanced stages of fibrosis.

### Implications for clinical practice and research

Our findings substantiate the growing concern about the aggressive clinical course of youth-onset T2D, demonstrating its impact on liver health. The high prevalence of MASLD in this group supports recommendations for early screening for this liver disease in the pediatric T2D population. However, given that a liver biopsy requires the consent of the child's caregiver and is not always feasible in clinical practice, there is an unmet need to develop and validate reliable, non-invasive tools (such as blood-based biomarkers) to assess liver fibrosis in this population [56]. These data are useful for informing pediatricians, diabetologists, hepatologists, and policymakers, raising awareness of the burden of MASLD in the pediatric T2D population. As pharmacological therapies for MASLD and T2D are developed for adults [57, 58], it is essential to translate these drugs for use in children. Future studies should investigate the epidemiology of MASLD in youth-onset T2D using the newly established nomenclature definition in diverse populations worldwide, explore how the MASLD-T2D phenotype impacts clinical parameters and long-term outcomes, and develop management algorithms to optimize the screening and treatment of these patients.

In summary, this systematic review and meta-analysis revealed that over one-third of pediatric patients with T2D have MASLD. Addressing liver disease in childhood is timely to alter the long-term trajectory of liver and cardiometabolic health. With the rising of pediatric T2D, integrating liver health into diabetes care pathways may be essential to reducing future complications and improving patient outcomes.

## Supplementary Information

Below is the link to the electronic supplementary material.

Supplementary file1 (DOCX 7645 KB)

## Data Availability

No datasets were generated or analysed during the current study.

## Abbreviations

MASLD: Metabolic dysfunction-associated steatotic liver disease
NAFLD: Non-alcoholic fatty liver disease
T2D: Type 2 diabetes
MRS: Magnetic resonance spectroscopy
MRI: Magnetic resonance imaging
T1D: Type 1 diabetes
