# Supplementary material for: The epidemiology of metabolic dysfunction-associated steatotic liver disease among pediatric patients with type 2 diabetes: Systematic review and meta-analysis
Source: Eur J Pediatr. 2026 Feb 5;185(2):120. doi: 10.1007/s00431-025-06734-0 (PMC12872636; doi:10.1007/s00431-025-06734-0)

**Supplementary Material**

**The Epidemiology of Metabolic Dysfunction-Associated Steatotic Liver Disease Among Pediatric Patients with Type 2 Diabetes: Systematic Review and Meta-Analysis**

**Summary of contents**

Supplementary Methods2

Supplementary Tables3

Supplementary Figures4

**Supplementary Methods – Search strategy**

Searched in PubMed and Embase databases from inception date to March 18, 2025:

| **PubMed** | ("type 2 diabetes"[All Fields] OR "T2DM"[All Fields] OR "T2D"[All Fields]) AND ("non-alcoholic fatty liver disease"[All Fields] OR ("naflds"[All Fields] OR "non-alcoholic fatty liver disease"[MeSH Terms] OR ("non alcoholic"[All Fields] AND "fatty"[All Fields] AND "liver"[All Fields] AND "disease"[All Fields]) OR "non-alcoholic fatty liver disease"[All Fields] OR "nafld"[All Fields]) OR (("metabolic"[All Fields] OR "metabolical"[All Fields] OR "metabolically"[All Fields] OR "metabolics"[All Fields] OR "metabolism"[MeSH Terms] OR "metabolism"[All Fields] OR "metabolisms"[All Fields] OR "metabolism"[MeSH Subheading] OR "metabolities"[All Fields] OR "metabolization"[All Fields] OR "metabolize"[All Fields] OR "metabolized"[All Fields] OR "metabolizer"[All Fields] OR "metabolizers"[All Fields] OR "metabolizes"[All Fields] OR "metabolizing"[All Fields]) AND ("dysfunctional"[All Fields] OR "dysfunctionals"[All Fields] OR "dysfunctioning"[All Fields] OR "dysfunctions"[All Fields] OR "physiopathology"[MeSH Subheading] OR "physiopathology"[All Fields] OR "dysfunction"[All Fields]) AND "steatotic"[All Fields] AND ("non-alcoholic fatty liver disease"[MeSH Terms] OR ("non alcoholic"[All Fields] AND "fatty"[All Fields] AND "liver"[All Fields] AND "disease"[All Fields]) OR "non-alcoholic fatty liver disease"[All Fields] OR ("fatty"[All Fields] AND "liver"[All Fields] AND "disease"[All Fields]) OR "fatty liver disease"[All Fields])) OR "MASLD"[All Fields] OR "metabolic-associated fatty liver disease"[All Fields] OR "metabolic dysfunction-associated steatotic liver disease"[All Fields] OR "MASH"[All Fields] OR "NASH"[All Fields] OR "non-alcoholic steatohepatitis"[All Fields] OR "metabolic dysfunction-associated steatohepatitis"[All Fields]) AND ("child*"[All Fields] OR "adolescent*"[All Fields] OR "teenager*"[All Fields] OR "pediatric*"[All Fields]) |
| --- | --- |
| **Embase** | ('type 2 diabetes'/exp OR 'type 2 diabetes' OR 't2dm'/exp OR t2dm OR t2d) AND ('non-alcoholic fatty liver disease'/exp OR 'non-alcoholic fatty liver disease' OR nafld OR masld OR 'metabolic-associated fatty liver disease'/exp OR 'metabolic-associated fatty liver disease' OR 'metabolic dysfunction-associated steatotic liver disease' OR mash OR nash OR 'non-alcoholic steatohepatitis' OR 'metabolic dysfunction-associated steatohepatitis') AND (child* OR adolescent* OR teenager* OR pediatric*) |

**Supplementary Table**

**Table S1 –** Meta-regressions showing the effect of potential moderators on MASLD prevalence in pediatrics with T2D. MASLD, metabolic dysfunction-associated steatotic liver disease; T2D, type 2 diabetes.

| **Moderator** | **Studies, n** | **Coefficient** | **95% confidence interval** | **p-value** |
| --- | --- | --- | --- | --- |
| Female, % | 15 | -0.025 | -0.059 to 0.010 | 0.158 |
| Mean age, years | 15 | 0.264 | -0.233 to 0.762 | 0.298 |
| Mean BMI-SDS | 11 | -1.102 | -2.414 to 0.211 | 0.100 |
|  | | | | |

**Supplementary Figures**

**Figure S1–** Traffic-light plots showing the risk of bias of the included studies.


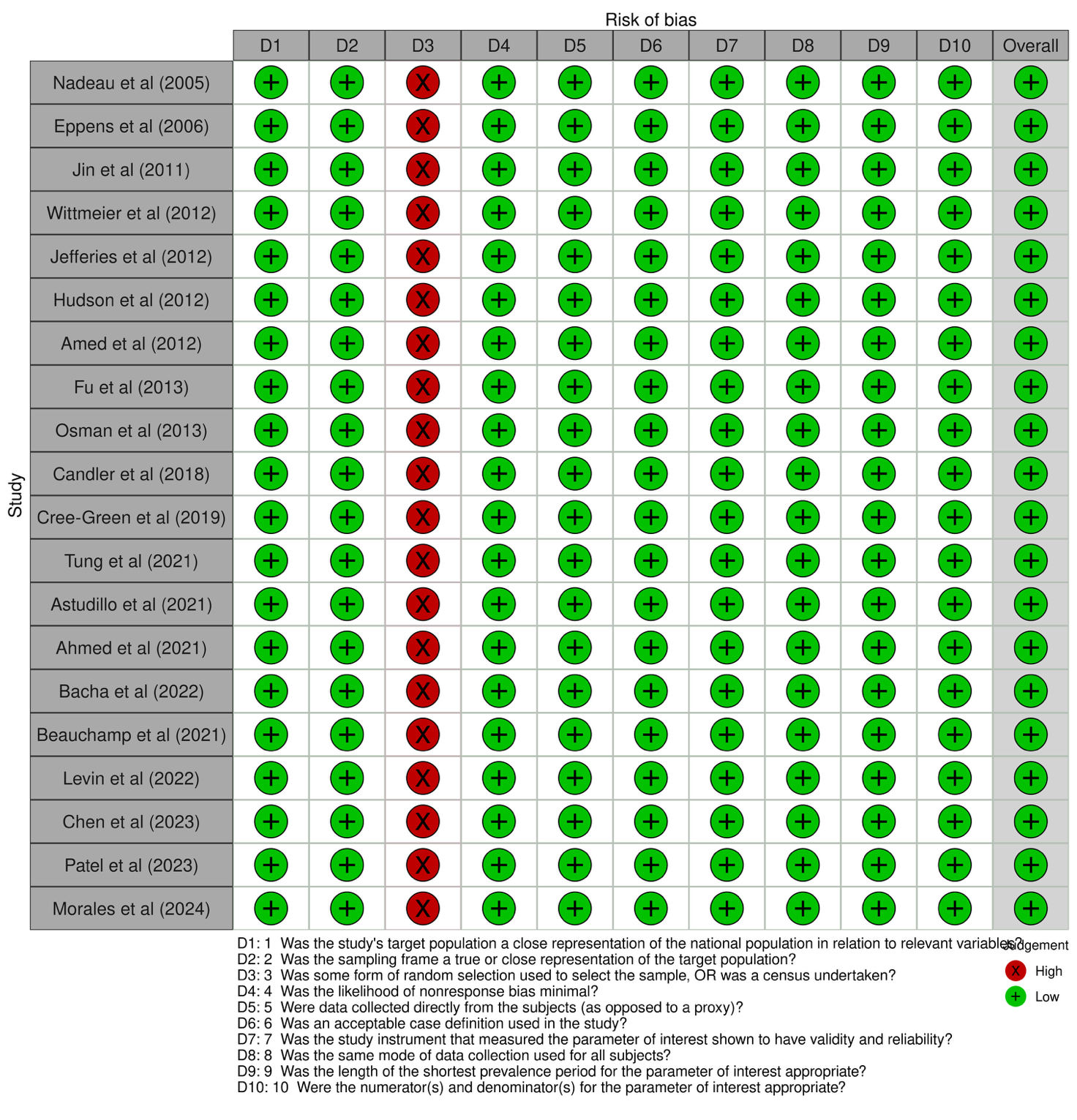


**Figure S2 –** Forest plot showing the sensitivity analysis restricted to imaging diagnosis.

**
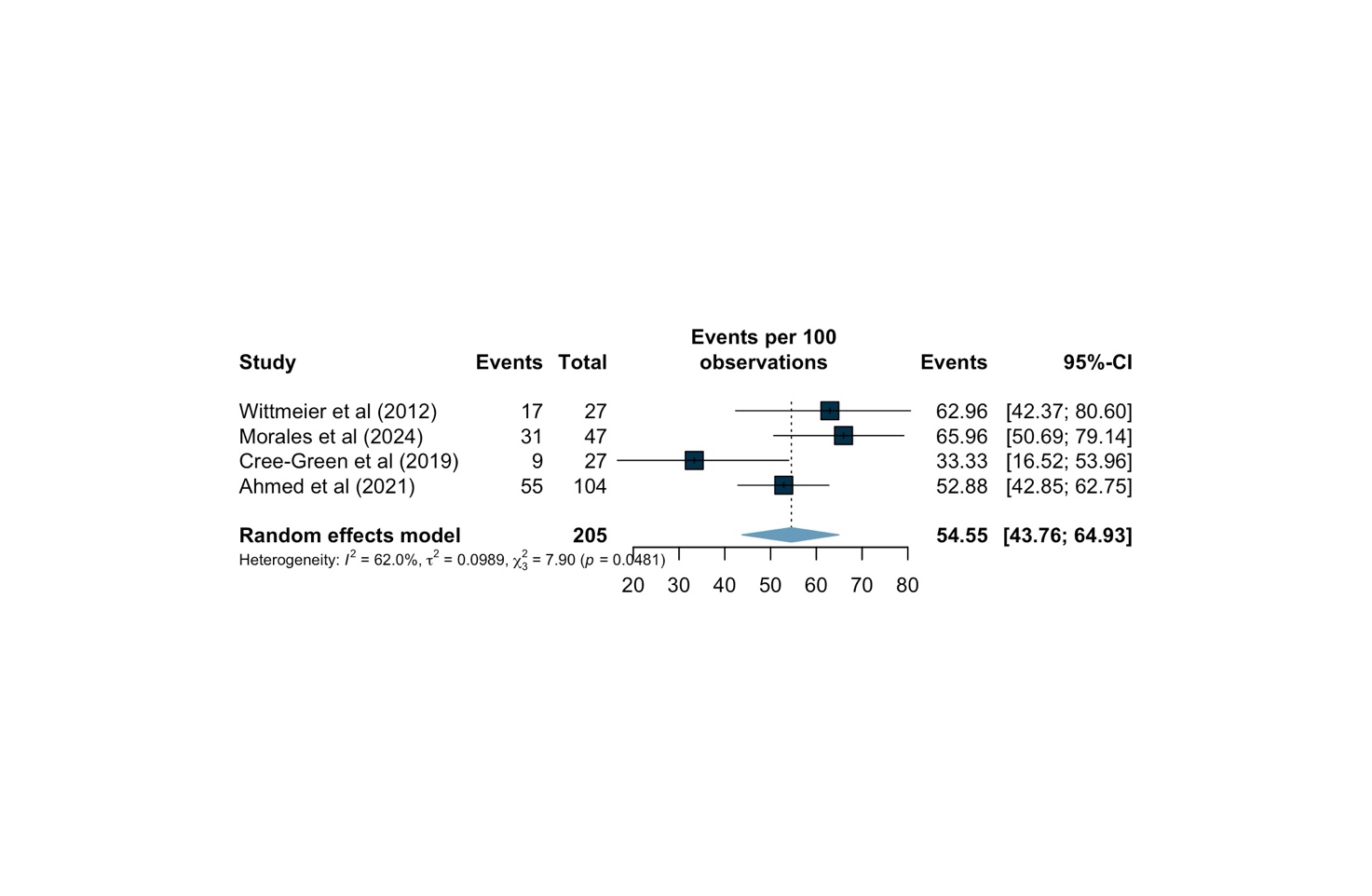
**

**Figure S3 –** Forest plot showing the sensitivity analysis restricted to MR-based diagnosis.


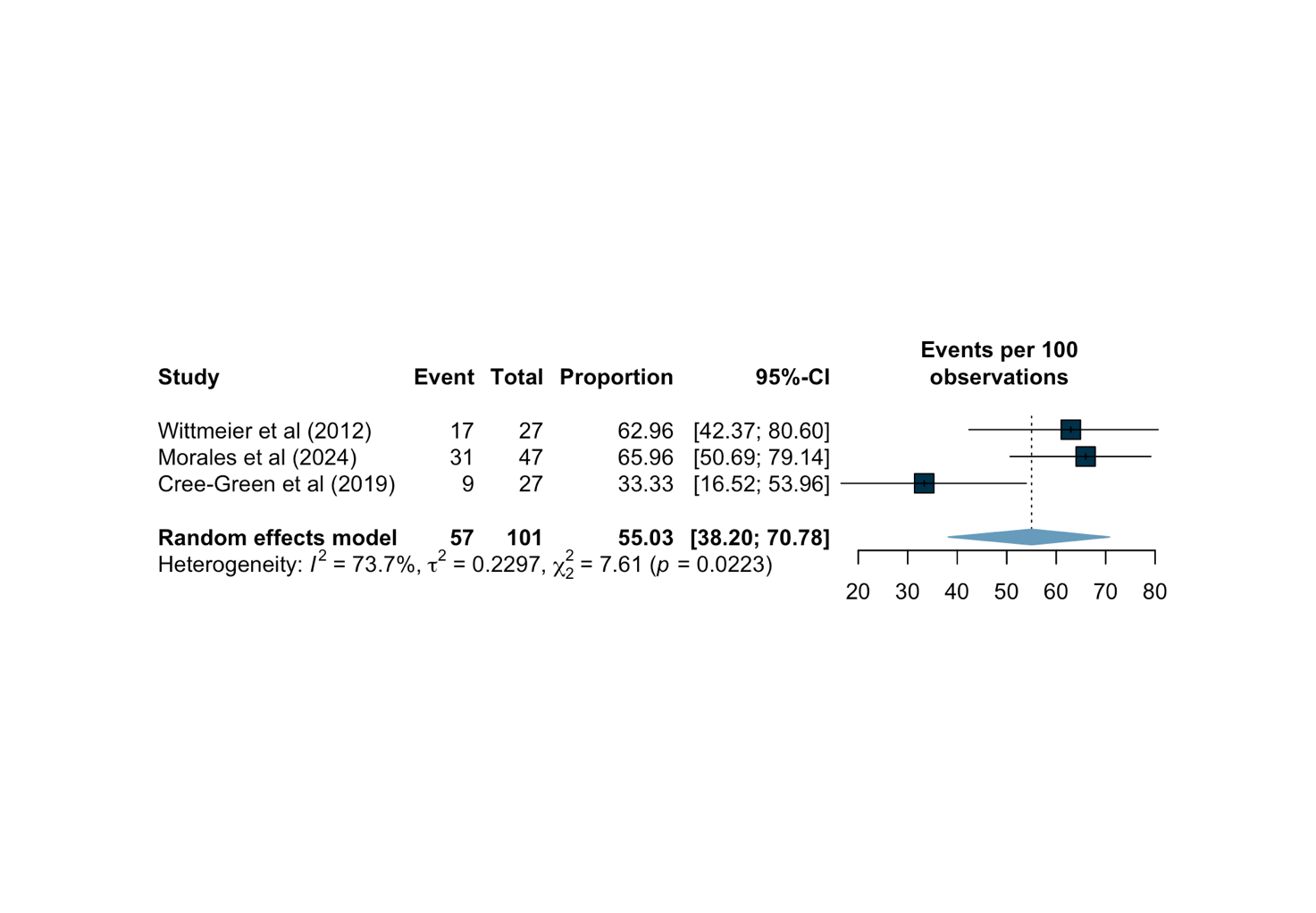

Supplement: Supplementary file 1 — Supplementary file1 (DOCX 7645 KB) [file 431_2025_6734_MOESM1_ESM.docx]
